# The impact of palliative care on quality of life, anxiety, and depression in idiopathic pulmonary fibrosis: a randomized controlled pilot study

**DOI:** 10.1186/s12931-019-1266-9

**Published:** 2020-01-03

**Authors:** Katherine Janssen, Drew Rosielle, Qi Wang, Hyun Joo Kim

**Affiliations:** 1 University of Minnesota Division of Pulmonary, Allergy, Critical Care, and Sleep Medicine, MMC 276, 420 Delaware Street SE, Minneapolis, MN 55455 USA; 2 University of Minnesota Palliative Care, MMC 603 Mayo, 8603A, 420 Delaware Street SE, Minneapolis, MN 55455 USA; 3 University of Minnesota Biostatistical Design and Support Center, Clinical Translational Science Institute, Room 223, 1932D, 717 Delaware St SE, Minneapolis, MN 55414 USA

**Keywords:** Idiopathic pulmonary fibrosis, Quality of life, Anxiety, Depression, Palliative care

## Abstract

**Background:**

Idiopathic pulmonary fibrosis (IPF) is a fatal disease that results in poor quality of life due to progressive respiratory symptoms, anxiety, and depression. Palliative care improves quality of life and survival in other progressive diseases. No randomized controlled trials have investigated the impact of palliative care on quality of life, anxiety, or depression in IPF.

**Methods:**

We conducted a randomized, controlled, pilot study to assess the feasibility of measuring the effect of a palliative care clinic referral on quality of life, anxiety, and depression in IPF. Patients were randomized to usual care (UC) or usual care + palliative care (UC + PC) with routine pulmonary follow up at 3 and 6 months. The UC + PC group received a minimum of one PC clinic visit. Primary outcome was change from baseline in quality of life, anxiety, and depression as measured by the St. George’s Respiratory Questionnaire (SGRQ), the Hospital Anxiety and Depression Index (HADS), and the Patient Health Questionnaire (PHQ-9) at 6 months.

**Results:**

Twenty-two patients were randomized between September 2017 through July 2018; 11 to UC and 11 to UC + PC. There was no difference in the change in SGRQ score at 3 months or 6 months, however, the symptom score trended towards a significant worsening for UC + PC at both 3 and 6 months (mean change at 3 months for UC and UC + PC was − 7.8 and + 10.7, respectively, *p* = 0.066; mean change at 6 months for UC and UC + PC was − 6.0 and + 4.6, respectively, *p* = 0.055). There was no difference in the change in HADS anxiety or depression scores. There was a significant transient worsening in PHQ-9 scores for UC + PC at 3 months (UC: -1.6, UC + PC: + 0.9, *p* = 0.008); this effect did not persist at 6 months.

**Conclusion:**

This pilot study demonstrated that a randomized controlled trial of palliative care in idiopathic pulmonary fibrosis patients is feasible. Receiving palliative care did not lead to improved quality of life, anxiety, or depression compared to usual care after 6 months. Patients in the UC + PC group trended towards worsening symptoms and a small but statistically significant transient worsening in depression. These findings should be interpreted with caution, and need to be evaluated in adequately powered clinical trials. NCT03981406, June 10, 2019, retrospectively registered.

## Introduction

Idiopathic pulmonary fibrosis (IPF) is a progressive fibrotic lung disease. The clinical course of IPF is variable and unpredictable, but universally fatal with a median survival of 2–3 years [1]. Patients with IPF experience significantly diminished quality of life due to worsening symptoms as the disease progresses. Recent data from the INSIGHTS-IPF registry demonstrated that quality of life is closely related to the clinical course of IPF [2]. In addition to worsening respiratory symptoms, fatigue, and deconditioning, patients with IPF experience significant depression and anxiety [3, 4]. There is a paucity of data addressing quality of life in IPF, and specific treatment guidelines do not exist [5–7].

The benefit of palliative care has been demonstrated in several other progressive diseases, most notably in metastatic lung cancer. Patients with metastatic lung cancer who were seen by palliative care at the time of their diagnosis and throughout their disease course were found to have increased survival, improved quality of life, and received less aggressive care at the end of their life [8]. This finding has prompted significant research in the role of palliative care in other diseases, including chronic lung disease. A non-blinded, randomized trial of a multi-disciplinary breathlessness support service for patients with cancer, chronic obstructive pulmonary disease (COPD), interstitial lung disease (ILD), and congestive heart failure (CHF) in the UK demonstrated improvement in breathlessness, anxiety, and even survival [9].

Patients with IPF experience less and later interaction with palliative care as compared with other patients [10–14]. When compared to patients with cancer, patients with chronic lung disease, including interstitial lung disease, are less likely to have a “do not resuscitate order”, and they die with increased breathlessness, unrelieved pain and anxiety [10, 11]. Referral to palliative care occurs in the minority of IPF patients. Multiple retrospective reviews have shown a palliative care referral rate between 3.8 to 13.7% [12–14], often within 1 month of the patient’s death.

Multiple qualitative studies and focused interviews have demonstrated significant physical and psychosocial needs in patients with IPF. This has prompted increased awareness of the role of palliative care and possible benefit in patients with IPF [15–19]. To date, few palliative care studies in IPF have examined quality of life as the primary outcome [20–23].

Despite the clear need for interventions to improve quality of life in patients with IPF, and potential benefits of palliative care in this disease, there is little evidence to guide therapies. Of the interventional studies which exist for IPF, few examine quality of life, anxiety, and depression as primary outcomes. Further, no randomized controlled trials exist to determine if receiving palliative care by palliative care providers improves quality of life or symptoms of depression or anxiety in patients with IPF [24, 25]. The goal of our study is to determine the feasibility of performing a randomized controlled trial to examine the effect of a structured palliative care clinic visit on quality of life in patients with IPF.

## Materials and methods

### Patient recruitment and randomization

Eligible patients were identified through the University of Minnesota ILD database and recruited in person during an ILD clinic visits, which typically occurred every 3 months. Eligible patients included patients ≥18 years with a diagnosis of IPF based on the 2011 international Evidence-based Guidelines [5]. Exclusion criteria included a documented malignancy that impacted mortality within the study period, inability to pay for the palliative care visit (via insurance or personally), and participation in another IPF clinical trial. Once informed consent was obtained, patients were randomized to usual care (UC) or usual care + palliative care (UC + PC) based on a permuted block design with random variable block sizes of 2 and 4.

### Primary outcomes

The primary outcomes for this study included change in respiratory quality of life, anxiety, and depression at 6 months. Respiratory quality of life was measured using the St. George Respiratory Questionnaire (SGRQ). The SGRQ is a 50-item disease specific questionnaire that has been validated for ILD [26–28]. The SGRQ asks questions with respect to 3 different domains including activity, symptoms, and impact. Scores range from 0 to 100, with a higher score indicating a worse quality of life. In ILD, The SGRQ has a minimum clinically important difference of 7 for the total score. The minimum clinically important difference for symptoms, activity, and impact scores are 8, 5, and 7, respectively [26]. Anxiety was measured using the Hospital Depression and Anxiety Scale (HADS). The HADS is a 14-item questionnaire which assesses symptoms of depression and anxiety independent of physical symptoms. The HADS generates a score for both depression and anxiety. Scores of 8 or higher for each category indicate a probable case of either depression or anxiety [29, 30]. Depression was measured using the HADS and the Patient Health Questionnaire (PHQ-9). A score of 5 or more on the PHQ-9 is considered mild depression [31].

### Secondary outcomes

Change in pulmonary function tests, number of hospitalizations, and all-cause mortality over 6 months were evaluated.

### Palliative care intervention

Patients randomized to UC + PC received a referral to the Palliative Care Clinic. The palliative care team is a multi-disciplinary group, including physicians, nurses, and social workers, who practice at a large, tertiary, academic medical center with a structured IPF and lung transplantation referral program. As a matter of policy, our palliative care team sees all patients undergoing lung transplant evaluation and has extensive experience with broad symptom management for advanced pulmonary diseases and end of life care. Patients in the UC + PC group received palliative care for a minimum of one outpatient visit between their baseline pulmonary visit and their 3 month follow up visit. The palliative care clinic intervention consisted of at least one visit each with a palliative care social worker and palliative medicine physician. More specific interventions and follow up were recommended based on this visit. A visit with palliative care addressed several topics including but not limited to introduction of the palliative care program and its role, comprehensive symptom and quality of life assessment, assessment of patient’s support network, assessment of patient’s understanding of their illness and prognosis, future planning decisions, and care goals. Patients were invited to discuss all topics, however, were not pressured to engage in discussions they did not feel prepared for.

### Follow up

Patients were followed over 6 months with study visits embedded into their regular IPF clinic visits including baseline visit, 3 month, and 6 month visits. Chart review occurred at each pulmonary clinic visit. Study questionnaires were completed by the study participants in clinic or online.

### Statistical analysis

Statistical support for this project was provided by the University of Minnesota Clinical and Translational Science Institute (CTSI).

Patient baseline demographic and clinical characteristics were summarized using descriptive statistics and were compared between groups using two-sample t test for continuous variables and Fisher’s exact test for categorical variables.

Pearson’s correlation coefficients were used to examine the correlation between pulmonary function tests and SGRQ overall, symptom, activity, and impact scores.

Intention to treat analysis was performed. Change in quality of life measures over time (from baseline to 3 months, and 6 months) were evaluated using repeated measures linear mixed models. Models included fixed effects of month (0, 3, or 6), treatment group, and month-by-treatment interaction, and a random intercept to account for correlations among repeated measures within patients. Rates of patient hospitalizations, death, and lung transplant were compared between treatment groups using Fishers exact test. Analyses were performed using SAS (Version 9.4, The SAS institute, Cary, NC). *P* values of less than 0.05 were considered statistically significant.

### Sample size and power

This is a pilot study and was not intended to be fully powered to demonstrate change. The data collected in this study will provide us estimates of the treatment effect to calculate the sample sizes for future studies.

## Results

### Patient characteristics

Fifty-five eligible patients were screened from September 2017 through July 2018. Of those patients, 22 consented to participate in the study. The mean age of the participants was 71.1 years (SD 7.6 years) (Table 1). Twenty patients were male, and 2 were female. The mean FVC of all the participants was 2.4 L, or 73.4% of predicted (SD 18.8%). The average duration of IPF diagnosis was 3.3 years (SD 3 years). (Table 1). Of the 33 patients who declined to participate in the study, 9 elected to enroll in other clinical trials, 2 requested a palliative care referral rather than participating in this trial, and 22 patients declined for various reasons.

**Table 1:** Demographic Characteristics

	Overall (*n* = 22)	Usual Care (*n* = 11)	Usual Care + Palliative Care (*n* = 11)
Gender, n (%)			
Male	20 (90%)	9 (82%)	11 (100%)
Female	2 (9%)	2 (18%)	0 (0%)
Age, mean (SD)	71.1 (7.6)	69.5 (7.2)	72.7 (8)
Smoking Status, n (%)			
Former	21 (95%)	11 (100%)	10 (90%)
Never	1 (5%)	0 (0%)	1 (9%)
Family History of IPF, n (%)			
No	16 (73%)	8 (72%)	8 (73%)
Yes	6 (27%)	3 (27%)	3 (27%)
Comorbidities^a^, n (%)			
No	1 (5%)	0 (0%)	1 (9%)
Yes	21 (95%)	11 (100%)	10 (90%)
Duration of IPF, mean (SD)	3.3 years (3)	3.6 years (2.4)	3.1 (3.6)
Antifibrotic Treatment, n (%)			
Pirfenidone	10 (45%)	6 (55%)	4 (36%)
Nintedanib	9 (41%)	4 (36%)	5 (45%)
FVC, percent predicted, mean (SD)	73.4% (18.8%)	72.9% (18.7%)	73.9% (19.8%)
FVC, L, mean (SD)	2.8 (0.7)	2.8 (0.8)	2.8 (0.6)
DLCO, percent predicted, mean (SD)	56.4% (13.8%)	54.9% (14.2%)	57.8% (13.9%)
DLCO, L, mean (SD)	14.1, (3.6)	13.7 (3.7)	14.5 (3.7)
TLC, L, percent predicted	68.9% (8.4%)	65.4% (7.2%)	71.4% (8.9%)
TLC, L, mean (SD)	4.8 (0.6)	4.5 (0.7)	5.1 (0.3)
6 min walk distance (*n* = 9)	540 m (SD 344.3)	584 m (SD 447.4), *n* = 4	504.8 m (SD 289), *n* = 5

^a^*CHF* Comorbidities include congestive heart failure, pulmonary hypertension, *OSA* Obstructive sleep apnea, *COPD* Chronic obstructive pulmonary disease, *DM* diabetes mellitus, and *CAD* Coronary artery disease

### Follow up and description of palliative care intervention

Eleven patients were randomized to the UC group, and 11 patients were randomized to UC + PC. In the UC group, 1 patient received a lung transplant and 1 patient died prior to 3-month follow up, and 9 patients completed 3 and 6-month follow up.

**Fig. 1:**
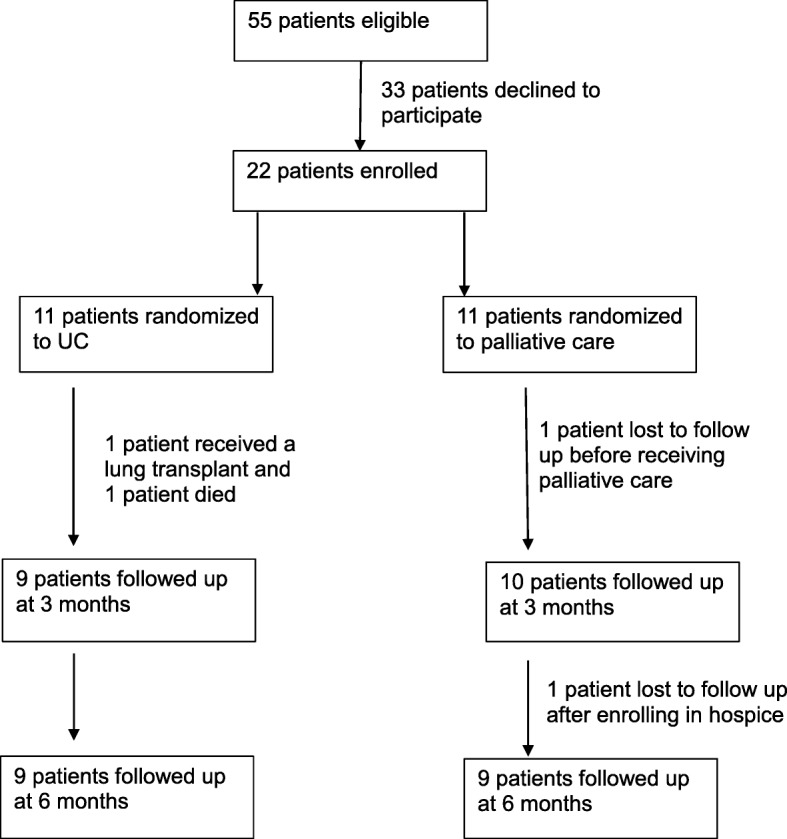
Patient recruitment and follow up

In the UC + PC group, 1 patient was lost to follow up prior to receiving palliative care and 3-month follow up. Ten patients received palliative care and followed up at 3 months. One patient enrolled in hospice and was lost to follow up between 3- and 6-month follow up. Nine patients in the UC + PC group completed 6-month follow up. (Fig. 1). Of the 10 patients in the UC + PC group, 6 patients saw the palliative care physician and social worker once, and 4 patients had 2 visits within the 6 month study period. Discussions included topics such as disease trajectory, current symptoms, mood, and advance care planning. Three patients filled out a Provider Orders for Life Sustaining Treatment (POLST) form. Two patients received pharmacologic interventions, and one was referred to hospice. Qualitative analysis of patient’s experiences in palliative care was not completed, however, informal comments from patients ranged from “I do not recall seeing these providers”, “this was completely unhelpful”, and “this was very helpful for me.”

### Baseline quality of life, anxiety, and depression

**Fig. 2:**
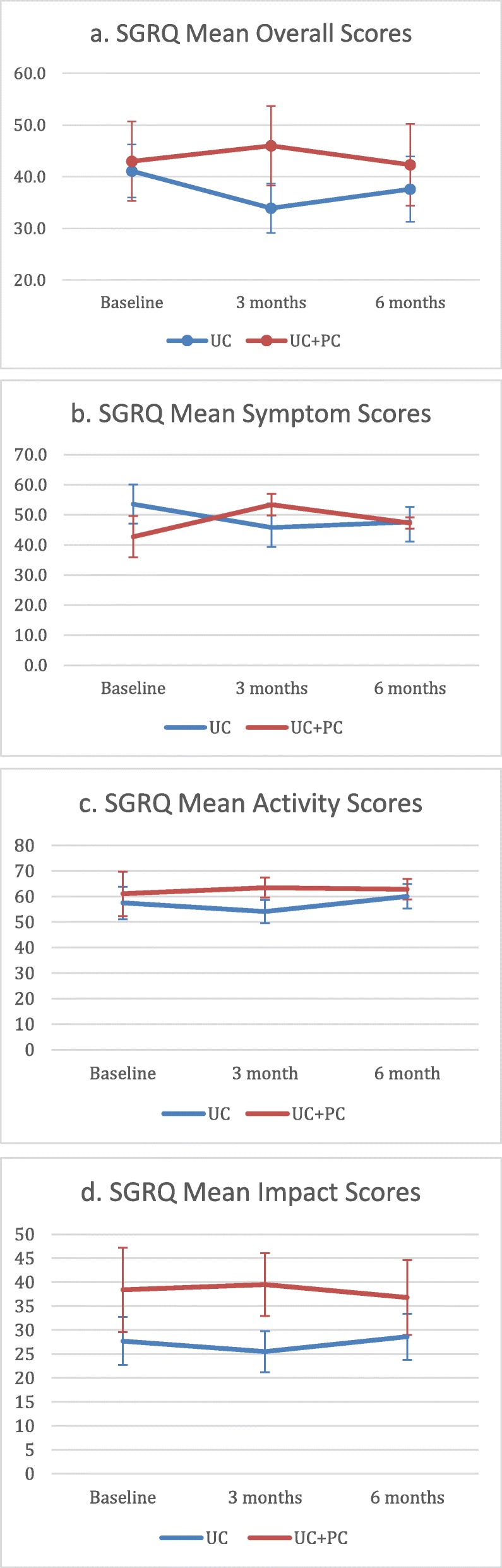
SGRQ scores plotted by time and treatment group, with standard error bars. **a** SGRQ Overall Score. **b** SGRQ Symptom Score. **c** SGRQ Activity Score. **d** SGRQ Impact Score

The overall mean SGRQ total score was 42, SD 20.3 (Table 2, Fig. 2). The mean scores for the activity, symptoms, and impact components were 59.3 (SD 24.7), 48.4 (SD 21.8), and 33 (SD 23.8), respectively. The mean baseline HADS anxiety score was 5.3 (SD 4.3). The mean baseline HADS depression score was 4.0 (SD 3.2). The mean baseline PHQ-9 score was 5.4 (SD 5.3). There was no significant difference in baseline scores for quality of life, anxiety, or depression between the two groups. The prevalence of depression by the HADS-D score (threshold of 8 or higher) was 9.09%, or 2/22 patients. The prevalence of depression by the PHQ-9 (threshold of 5 or higher) was 36.4% or 8/22 patients. Most of these (6/8, 75%) were considered mild depression. The prevalence of anxiety by the HADS-A score (threshold of 8 or higher) was 36.4%, or 8/22 patients.

**Table 2:** Primary Outcomes and change in pulmonary function tests: Baseline mean SGRQ, HADS, and PHQ-9 scores and pulmonary function tests with mean change at 3 and 6 months

	Baseline		3 months			6 months		
	UC *N* = 11 Mean, (SD)	UC + PC *N* = 11 Mean, (SD)	UC *N* = 9Mean Change, (SD)	UC + PC *N* = 9Mean Change, (SD)	*p*	UC *N* = 9Mean Change, (SD)	UC + PC *N* = 9Mean Change, (SD)	*p*
SGRQ Total	41.1 (16.8)	43 (24.4)	−3.6 (10.3)	+ 5.2 (12.7)	0.097	+ 0.1 (12.3)	+ 3.8 (11.9)	0.44
SGRQ Impact	27.7 (16.6)	38.4 (29.1)	−2.2 (12.8)	+ 1.1 (19.6)	0.69	+ 0.9 (14.4)	−1.6 (23.4)	0.99
SGRQ Activity	57.5 (21.1)	61.1 (28.8)	−3.4 (13.6)	+ 2.4 (11.7)	0.32	+ 2.6 (14.4)	+ 1.8 (12.1)	0.94
SGRQ Symptoms	53.6 (21.6)	42.7 (21.7)	−7.8 (23.9)	+ 10.7 (10.6)	0.066	−6.0 (15.2)	+ 4.6 (5.5)	0.055
HADS(A)	4.3 (3.3)	6.3 (5.1)	−0.4 (2.9)	+ 0.3 (2.4)	0.59	+ 0.4 (3.2)	−0.8 (2.3)	0.35
HADS(D)	4.1 (2.9)	3.9 (3.6)	−0.3 (2.5)	+ 0.4 (0.0)	0.49	+ 0.7 (1.9)	+ 0.7 (2.2)	0.99
PHQ-9	6.5 (3.3)	4.4 (6.8)	−1.6 (1.7)	+ 0.9 (1.8)	0.008	−0.7 (3.5)	+ 2.1 (2.4)	0.26
FVC (% predicted)	72.9% (18.7%)	73.9% (19.8%)	+ 1.5% (5.1%)	+ 1.0% (6.4%)	0.83	+ 0.2% (6.0%	−4.1% (9.0%)	0.36
TLC (% predicted)	65.4% (7.2%)	71.4% (8.9%)	+ 3.3% (9.2%)	−7.0% (3.0%)	0.040	+ 1.3% (6.7%)	−14.7% (4.5%)	0.001
DLCO (% predicted)	54.9% (14.2%)	57.8% (13.9%)	−1.4% (5.4%)	−5.1% (7.0%)	0.34	−4.0% (5.3%)	−2.3% (6.5%)	0.73

### Quality of life, anxiety, and depression at 3 months

There was no significant change in SGRQ total score, impacts, or activity scores between the UC and UC + PC groups at 3-month follow up (Table 2, Fig. 2). The SGRQ Symptom score worsened in the UC + PC group but not the UC group; − 7.8 (SD 23.9) for UC, and + 10.7 (SD 10.6) for the UC + PC group (*p* = 0.066)). This trended towards statistical significance and was higher than the minimum clinically important difference of 8 for ILD. There was no significant difference in the HADS anxiety or HADS depression scores. There was a significant worsening of the PHQ-9 score for the UC + PC group as compared to UC group (− 1.6, SD 1.7 for UC, + 0.9, SD 1.8 for UC + PC, *p* = 0.008).

### Quality of life, anxiety, and depression at 6 months

There was no significant difference in the change in SGRQ total score, impacts, or activity scores between the UC and UC + PC groups at 6 months (Table 2, Fig. 2); The change in symptom score was − 6.0 points (SD 15.2) for UC, and + 4.6 points (SD 5.5) for UC + PC (*p* = 0.055). This trended towards statistical significance but did not meet the minimum clinically important difference of 8 for ILD. There was no significant difference in the change in HADS anxiety or HADS depression scores, and no significant difference in the change in PHQ-9 scores.

### Secondary outcomes

There was no significant change in forced vital capacity (FVC), forced expiratory volume in 1 s (FEV1), or diffusing capacity (DLCO) in the UC or UC + PC group at 3 months or 6 months (Table 2). However, there was a statistically significant worsening of the total lung capacity (TLC) in the UC + PC group at both 3 months and 6 months. At 3 months, the change in TLC for UC was + 3.3% (SD 9.2%), and the change for UC + PC was − 7.0% (SD 3.0%,) *p* = 0.04. At 6 months, the change in TLC for UC was + 1.3% (SD 6.7%) and − 14.7%, (SD 4.5%) for UC + PC, *p* = 0.001. There was no significant difference in patient hospitalizations, death, or lung transplant (Table 3).

**Table 3:** Hospitalizations, deaths, lung transplant at 3 months and 6 months

	3 month			6 month		
	UC *N* = 9	UC + PC *N* = 9	*P* value	UC *N* = 9	UC + PC *N* = 9	*P* value
Hospitalized, n (%)	3 (27)	0 (0)	0.21	2 (22)	2 (22)	1
Death, n (%)	1 (9)	0 (0)	1	0 (0)	0 (0)	1
Lung Transplant, n (%)	1 (10)	0 (0)	1	0 (0)	0 (0)	1

### Correlation of pulmonary function tests and quality of life

The TLC, FVC, and DCLO were all negatively correlated with the SGRQ overall, symptom, impact, and activity scores. The correlation between the TLC percent predicted and SGRQ overall, symptom, impact, and activity scores were − 0.64, − 0.55, − 0.58, and − 0.59, respectively; *p* < 0.0001. The correlation between FVC percent predicted and SGRQ overall, symptom, impact, and activity scores were − 0.46, − 0.52, − 0.36, and − 0.45, respectively; *p* < 0.0001. The correlation between the DLCO percent predicted and SGRQ overall, symptom, impact, and activity scores were − 0.64, − 0.42, − 0.60, and − 0.66, respectively; *p* < 0.0001.

### Barriers to enrollment

While a qualitative analysis of reasons for declining participation was not completed, eligible patients raised several issues. These included logistical issues frequently encountered in clinical trials such as lack of interest in extra visits, finding parking, and time needed to fill out questionnaires. Other patients elected to participate in other clinical trials. Patients’ presumably preconceived ideas of palliative care also played a role in enrollment as well. After hearing the study description, 2 patients requested to see palliative care outright. Despite clarification of the role of palliative care, other patients were concerned that seeing palliative care was a step closer to hospice care, and felt this was unnecessary at their stage in the disease.

## Discussion

This study was a randomized, controlled pilot study which demonstrated the feasibility of completing a larger trial to examine the effect of palliative care in patients with IPF. Of the 22 patients enrolled, 18 patients (81.8%) completed the study. Multiple barriers to enrollment in a study of this nature were identified during patient recruitment. Regardless of disease severity, patient attitudes and understanding of palliative care could result in selection bias. This will need to be addressed prior to conducting future studies.

While our results need to be interpreted with caution, our findings suggest that receiving palliative care may cause a worsening symptom-related quality of life in IPF in the short term and a possible transient worsening of depression.

There are a few potential explanations as to why receiving palliative care could worsen quality of life in the short term. Our study population had mild disease, with an average FVC of 73.4% predicted. It is possible that patients received palliative care too early in their disease course, and discussions regarding prognosis may have worsened symptoms of depression or anxiety. Additionally, receiving a structured palliative care visit may have increased patients’ awareness and/or perception of their symptoms and disease prognosis. This finding is not dissimilar to the study conducted by Lindell and colleagues in 2010, in which IPF patients who went to a series of 6 support group sessions led by a clinical nurse specialist, a psychiatric clinical specialist, and an advanced care planning instructor. Patients randomized to the support group actually had reduced health related quality of life and increased anxiety, but improved satisfaction with participation in the support group [20]. Conversely, Bajwah and colleagues completed a pilot randomized controlled trial in the UK demonstrating that a multidisciplinary palliative case conference intervention improved patient quality of life parameters compared to usual care [21]. This intervention included a case conference led by a palliative care nurse specialist, and attended by the patient, their caregiver (if able), and a community palliative care nurse. This approach did not offer palliative care directly to patients in a clinical setting. A before and after study of an integrated early palliative care approach demonstrated reduced hospital utilization within the last year of life and increased number of home deaths [22], but patient quality of life was not assessed. IPF patients also may require more longitudinal follow up with palliative care prior to experiencing benefit.

Alternatively, routine palliative care referrals may be an inefficient way to address the palliative care needs of patients with IPF. IPF is a rare disease, and, while this is not the case at our institution, not all palliative care physicians may be familiar with the clinical course and prognosis, necessitating a more tailored palliative care approach specific for IPF patients. Other studies in chronic disease demonstrated the benefit of a multi-disciplinary approach [9, 21]. A large randomized controlled trial of a multi-disciplinary intervention (SUPPORT) for IPF patients and their caregivers is ongoing. Primary outcomes will include quality of life, symptom burden, and stress burden, among others [23].

Interestingly, the prevalence of baseline depression was much lower based on the HADS score as compared to the PFQ-9 (9 vs 36%). This difference may reflect the nature of the different questionnaires. The finding of worsening TLC in the UC + PC group, though statistically significant, cannot be explained, and could be due to the small number of participants in this trial.

Our study also identified a negative correlation between pulmonary function testing and quality of life as measured by the SGRQ. This is similar to findings from other IPF registry studies [2, 32].

Our study is limited due to the nature of our single center study and small number of patients. Selection bias based on patients’ attitudes and understanding of palliative care is likely an issue as well. A larger randomized trial is necessary to confirm these findings. The palliative care intervention itself is difficult to standardize and heterogeneous, as each visit depends on each patient’s unique needs. Other limitations include the challenging task of assessing patients’ quality of life, depression, or anxiety. Several instruments have been validated to assess health related quality of life in ILD, however, one is not clearly utilized more than the other. Multiple instruments may be necessary in future studies; we selected the SGRQ due to its familiarity, and desire to limit the number of surveys filled out by the patients. Measuring anxiety and depression is also challenging as several instruments exist to measure both mood disorders, and are also used heterogeneously in the literature.

## Conclusion

In IPF, a randomized controlled trial to measure the impact of palliative care on quality of life, anxiety, and depression is feasible. It is possible that receiving palliative care could trend toward a worsening symptom-related quality of life at 6 months, as measured by the SGRQ, and a possible transient worsening of depression, however, larger randomized trials are necessary to determine if this is a true effect. Further trials should focus on reducing selection bias in participants, the optimal timing of palliative care, and which questionnaire(s) best captures depression and anxiety.

## Data Availability

The datasets generated and analyzed during the current study are available from the corresponding author on reasonable request.

## Abbreviations

CHF: Congestive heart failure
COPD: Chronic obstructive pulmonary disease
DLCO: Diffusing capacity of the lungs for carbon monoxide
FEV1: Forced Expiratory Volume in 1 s
FVC: Forced Vital Capacity
HADS: Hospital Anxiety and Depression Index
HADS-A: Hospital Anxiety and Depression Index-Anxiety score
HADS-D: Hospital Anxiety and Depression Index-Depression score
ILD: Interstitial lung disease
IPF: Idiopathic pulmonary fibrosis
PHQ-9: Patient Health Questionnaire
SGRQ: St. George’s Respiratory Questionnaire
TLC: Total Lung Capacity
UC + PC: Usual care + palliative care
UC: Usual care
